# Camel production systems in Ethiopia: a review of literature with notes on MERS-CoV risk factors

**DOI:** 10.1186/s13570-018-0135-3

**Published:** 2018-12-21

**Authors:** Tadele Mirkena, Elias Walelign, Nega Tewolde, Getachew Gari, Getachew Abebe, Scott Newman

**Affiliations:** 1 Emergency Centre for Transboundary Animal Diseases, Food and Agriculture Organization of the United Nations, Addis Ababa, Ethiopia; 2 Regional Office for Africa, Food and Agriculture Organization of the United Nations, Accra, Ghana

**Keywords:** Ethiopia, Dromedary camel, Production system, Pastoralism, MERS-CoV

## Abstract

Camels are the most adapted species to the harsh conditions of arid/semi-arid rangelands of Ethiopia where pastoralism is the dominant mode of life and mobility is an inherent strategy to efficiently utilize the spatially and temporally distributed pasture and water resources. Usually, large numbers of camels and other domestic animals from many different herds/flocks congregate at watering sites, and this may create a perfect condition for disease transmission and spread among animals. The same water sources are also shared by multitudes of wild animals. Camel herd sizes per household range from few heads (five to ten) to several hundreds. Female camels account for more than 75% of the herd. Male camels are usually sold early as pack animals or for slaughter. Female camels may remain fertile up to 25 years, during which time they produce eight to ten calves. Camels are herded during daytime on communal rangelands. During night, they are kept in traditional *kraals* around homesteads. Breeding time is short and seasonal and is affected by rainfall patterns and feed availability. Usually, only men milk camels. Milking frequency ranges from two to five times per day. Washing of hands, milking vessels, the udder and teats is not practised by many prior to milking the camels. Besides, the milking area is generally full of dust and dung and without shade. This affects the quality and safety of the produced milk. Pathogens and diseases of camelids are less well known; however, they are suspected as zoonotic sources for the human infection with the Middle East respiratory syndrome coronavirus. There is an increasing need to determine whether camels are clinically susceptible, act as potential reservoirs and maintenance or bridge hosts, to viral pathogens.

## Introduction

The one-humped camel (*Camelus dromedarius*) is an important livestock species uniquely adapted to hot and arid environments. It produces milk, meat, wool, hair and hides and serves for riding, as a beast of burden and draught animal for agriculture and short-distance transport (Schwartz and Walsh 1992). The global population of domesticated large camelids (dromedaries and Bactrian) is estimated to be about 28 million (Faye 2015). This number is probably underestimated particularly in the Sahelian countries (Mauritania, Mali, Niger, Chad and Sudan) and Ethiopia. More than 80% of the camel population inhabits Africa with 60% in the Eastern African countries (Sudan, Somalia, Ethiopia and Kenya) which are important exporters of dromedary camels to the Arabian Peninsula and Egypt (Faye 2015). The camel population in Ethiopia is estimated at 4.8 million (Behnke 2010).

Major camel-keeping societies in Ethiopia include Afar, Somali, Oromo (Karayu, Gabra, Boran and Guji groups), Kunama and Irob peoples, among others. The Afar and the Somali peoples are known for their camel-keeping traditions for centuries; the Boran and Guji pastoralists, on the other hand, started camel production recently. Gabra and Somali, who have been keeping camels for centuries, are believed to play instrumental roles in introducing camels to the Borana Plateau (Coppock 1994). Besides, farmers and agro-pastoral communities in mid-altitude areas who were not traditionally camel keepers recently started adopting camels. Consequently, one can nowadays see camels along a vast expanse of central, north-eastern and north-western mid-altitude regions of the country (Aklilu and Catley 2011). Nearly all the camels sold by mid-altitude farmers and traders are male, essentially because of three reasons: market demand for male camels, female camels are never used for loading, and most pastoralists do not sell female camels (Aklilu and Catley 2011).

Drivers of global disease dynamics are believed to comprise human demographics, pressures on land and water resources, increased mobility, trade and transport volumes, climate change, deforestation and general degradation of natural ecosystems (Slingenbergh 2016). In September 2012, the first case of a human infected by a novel coronavirus, the Middle East respiratory syndrome coronavirus (MERS-CoV), was identified in Saudi Arabia. By September 2017, 2,091 cases of MERS-CoV have been reported to the World Health Organization, with at least 779 deaths, mostly in Saudi Arabia but also in 27 other countries (WHO 2017). Dromedary camels are the suspected zoonotic sources. Cases of MERS-CoV associated with travel or residence in the Near East have been reported from many other countries around the world, but the first large outbreak outside the Arabian Peninsula occurred in the Republic of Korea and China, with 189 cases and 36 deaths. The index case in this outbreak was known to have travelled to the Near East (Miguel et al. 2016).

The objective of this review was to collate information on camel production systems and lifestyles, husbandry practices and risky behaviours that potentiate MERS-CoV zoonotic transmission and spread among camels and to people in Ethiopia.

## Camel production environments and production systems

### Description of the production environments

The Ethiopian arid/semi-arid rangelands, where land use options such as agriculture are not economically and ecologically feasible, are estimated at about 78 million ha of land and cover close to 61% of the total landmass (EPA 1998, cited by Bruke 2003). The environment is a basic determinant of the nature and productivity of the range ecosystem. Physical factors such as climate, topography and soil determine the potential of the rangeland to support certain types and levels of land use (Tefera and Abebe 2012). In Ethiopia, the lowlands were traditionally defined as areas below 1,500 m above sea level (m.a.s.l.) and are classified into arid, semi-arid and sub-humid agro-ecological zones. These areas form the major rangelands of the country which are home to the pastoral and agro-pastoral communities and hence have primarily been used for livestock production. These areas are characterized by high ambient temperature, low rainfall, sparse vegetation and hence scarce feed resources. The mean maximum and mean minimum temperatures are 35 °C and 27 °C, respectively, with the hottest day in areas like Afar surpassing 45 °C (Tefera and Abebe 2012). They receive low and erratic rains that occur with highly seasonal patterns (either unimodal or bimodal) - for instance, a maximum of 250-mm rain may be received per year along the eastern Ethiopian border. The amount of rain is highly associated with and determines rangeland vegetation pattern.

The Ethiopian lowland is home to about 12% of the country’s human population. Approximately 93% of these are pastoralists and agro-pastoralists (Bruke 2003). In terms of livestock, the rangelands carry about 28% of the cattle, 60% of the goats, 26% of the sheep and almost all the camels representing about 26% of the total livestock resource base of the country. Lowland breeds of livestock significantly contribute to the national economy as the main source (~ 90%) of export animals and animal products (mainly meat and leather). The overwhelming volume of meat consumed domestically is also produced from animals reared in the lowlands. Camels are the most adapted species to the harsh conditions of the arid and semi-arid environments and can survive, reproduce and produce milk when and where other livestock species fail.

The Ethiopian rangelands currently face several threats. The notable threats are expansion of sedentary smallholder agriculture, large-scale agricultural and hydro projects, establishment of wildlife parks/sanctuaries, encroachment by invasive vegetation, intra- and inter-clan conflicts, droughts and deterioration of traditional institutions (Bruke 2003; Gebru et al. 2008; Tadesse et al. 2015a, b). Expansion of sedentary agriculture and large-scale irrigated agricultural projects, besides diminishing the existing traditional pastoral territories, can have significant impact on the ecology and welfare of downstream inhabitants. In Borana, community responses to changing land use resulted in the development of range enclosures, the expansion of crop farming and fragmentation of communal rangelands, while the suppression of fire contributed to expansion of bush encroachment (further detail on the latter is given below). The overall impact has been forage scarcity and greater vulnerability of livestock during drought years (Angassa and Oba 2008). In the past, the lower limit for sedentary agriculture and the upper limit for the rangelands were considered to be the escarpments receiving 500 to 700 mm of annual rainfall. Areas found in this range and below are actually marginal for rain-fed agriculture. However, due to population pressure and overexploitation of croplands in the adjacent highland areas, the rangelands are being encroached by sedentary crop cultivators.

In an effort to diversify livelihoods, some pastoralists have been venturing into crop cultivation. According to a report on land use/cover of the pastoral regions more than a decade ago, areas categorized or converted to crop cultivation showed drastic change (Bruke 2003). These include 178,000 ha in the Afar, 390,000 ha in Somali, 1,332,000 ha in the Borana zone of Oromia, 58,803 ha in south Omo, 32,452 ha in Gambela and 38,717 ha in Beneshangul Gumz. Using this crude estimate, the total area of the rangelands converted into crop agriculture could be in the range of 1.9 million ha. Only less than 2% of the Borana plateau was under small-scale cultivation in 1986 following the 1983/1984 drought, and this was limited to bottomlands and upland sites in the sub-humid and upper semi-arid zones (Coppock 1994).

Encroachment by invasive species is also a major threat to the livelihoods of pastoral communities. In Borana, encroachment of unwanted woody plant species increased after the 1960s and worsened following a ban on the use of fire (Angassa and Oba 2008). According to the authors, Boran pastoralists perceived that the result of the official ban on fire was a shift in vegetation composition from perennial grassland to bush encroachment and that it became severe about two decades after the official ban of range fires. Coppock (1994) reported that about 16 woody plant species are considered to be encroachers in the Borana rangeland. By mid-1980s, about 40% of the rangeland was already lost to bush encroachment (Coppock 1994). Only about 10% of the remaining area was considered to be in good condition and reserved for calves (Oba 1998, cited by Bruke 2003). Among the different species, rapid expansion of *Acacia drepanolobium* is the most alarming (Bruke 2003). In Afar and some areas of Somali regions, rapid expansion of *Prosopis juliflora* is a prime concern. Besides aggressively claiming prime irrigable and pasturelands, its use as livestock feed is negligible except a limited attempt made to investigate utilization of its pods as animal feed. In the Somali region, the rapid expansion of *parthenium* into the rangelands and crop farms is also alarming (Bruke 2003). These invasive plant species reduce the size of the usable rangelands, poison and kill animals when consumed and have a negative effect on the composition and taste of milk (Admasu 2008; Shackleton et al. 2014).

Conflicts and recurrent droughts limit use of rangeland resources. Intra- and inter-clan conflicts over the rangeland resources, mainly grazing and water, are common in and across all rangeland ecologies. Such conflicts usually contribute to a decline and overuse (sometimes underuse) of the resources. The recurrence and severity of drought intensified in the past five to six decades. Pastoralists faced five to seven drought periods during the past 30 years and lost 45 to 70% of their cattle in each of the periods. Because of the 2001/2002 drought, for instance, average livestock holding dropped from 60 to 26 and from 26 to 11 heads of cattle per household for Afar and Oromo pastoralists, respectively, living around the Awash National Park (Gebru et al. 2008). Drought resulted in 58% of cattle losses during this crisis period. Some of the pastoralists’ coping strategies are mobility/transhumance and diversification of livestock species. The change in vegetation composition coupled with climatic variability forced pastoralists to spread the risk they face by raising different but easily adaptable species. For instance, Afar pastoralists in the past preferred to raise cattle; they now prefer to raise camels followed by small ruminants and cattle (Gebru et al. 2008). The Somali pastoralists prefer to keep camels followed by small ruminants and cattle. In Borana, camel rearing next to cattle has become popular (Megersa et al. 2008).

Establishment of several parks and wildlife reserves in various parts of the country, predominantly in the rangeland ecosystem, has greatly restricted access of pastoralists and their livestock to prime grazing lands. In Afar region alone, there are about eight national parks, wild reserves and controlled hunting areas with a total land area of 353,730 ha (these are Awash and Yangudirassa national parks; Halaidegi, West Awash, Gewane and Mille Serdo wildlife reserves; and Gewane and West Awash controlled hunting areas). There are more than 81 species of mammals and 453 species of birds (6 of them endemic) in Awash Park alone (Gebru et al. 2008). According to informants contacted by Gebru et al. (2008), lions were predominantly found outside the park boundary, living in bush-encroached areas near human villages. A lion may kill three to five cattle per day in Awash Fentale district. The pastoralists (53% and 76% of Oromo and Afar, respectively) indicated that predators had killed their animals (Gebru et al. 2008).

Dwindling natural resources such as pasture and water has already forced the traditionally pastoral communities not only to change species composition of their livestock but also engage in other petty cash-earning activities. For instance, in the past, the Karayu pastoralists were heavily dependent on cattle. The increase in number of camels among the Karayu is a recent phenomenon that has been the direct consequence of ecological change and the inability of cattle to cope with the diminishing pasture and water resources (Gebru et al. 2008). Pastoralists also engage in off-farm activities such as selling firewood and charcoal, and petty trading to diversify income. Accordingly 15 to 35, 20 to 25 and 5 to 10% of pastoralists and agro-pastoralists in Somali, Borana and Afar, respectively, were engaged in different off-farm activities (Tadesse et al. 2013).

### Camel production systems

Livestock production systems in Ethiopia can be broadly classified into two as the traditional production systems (pastoral nomadic, pastoral transhumant, agro-pastoral and smallholder mixed crop-livestock) and the modern production systems (ranching, intensive/semi-intensive peri-urban/urban, feedlot and commercial production). Camels are predominantly kept in the pastoral and agro-pastoral production systems. Only few male camels are to be found in the mixed crop-livestock system.

Pastoralists keep indigenous breeds/types and obtain more than 50% of household income from livestock and livestock products. The system is much simpler than the mixed crop-livestock systems of the highlands. There are few inputs other than labour. Herd and flock composition is regulated to some extent (only few breeding males are maintained). Grazing management and herd movement are determined by the seasonal patterns of rainfall and availability of water. There is little to no interaction with crop agriculture, and although a range of livestock species is managed to reduce risk, one or two species dominate. For example, camels and goats are the main species in Afar and Somali, while in Borana zone, cattle are still the main species. Production is mainly for subsistence, but surplus animals are sold. Generally, camel populations have been increasing in the pastoral areas during the past 20 years by at least 10, 20, 25, 15, 25 and more than 200% in Gode, Jijiga, Shinille, Mille, Amibara districts and Borana zone, respectively. On the contrary, cattle populations decreased by 50 to 70% in these districts during the same time (Tadesse et al. 2013). According to these authors, about 14, 25, 10 and 8% of the households studied in Gode, Jijiga, Shinille and Borana, respectively, do not possess cattle at present. In the agro-pastoral production system, crop agriculture is combined to a limited extent with livestock rearing. It is practised in semi-arid areas and may take the form of either sedentary or transhumance way of living. Indigenous breeds/types are reared and livestock contribute between 10 and 50% of household incomes. Mixed crop-livestock production systems prevail in sub-humid and humid central highland parts of Ethiopia. The system is sedentary and livestock is secondary to crop production. It is characterized by smallholdings of about 1 to 3 ha of land and two to four heads of cattle (MoARD 2007).

### Feed and water resources

The major feed resources for camels are browsing trees or bushes, but grasses may be consumed when shrubs or trees are not available. The browse species are mostly leguminous trees and shrubs and many being salt bush plants of the family *Chenopodiaceae* and similar families (Wilson 1989; Bekele and Kibebew 2002). Feeds selected by camels are usually high in moisture, nitrogen, electrolytes and oxalates. Based on their preference, the most important plants browsed by camels are *Acacia brevispica*, *Opuntia ficus indica* and *Dichrostachys ciniarea*. These form important constituents of the dromedary diet wherever they are found (Wilson 1989; Bekele and Kibebew 2002). Camels also favourably feed on *Euphorbia tirucalli* and cacti (*Opuntia ficus indica*) and various crop residues (Aklilu and Catley 2011).

Wells, ponds and rivers are the main sources of water for camels (Wolde 1991; Coppock 1994). Watering animals from the deep wells is an arduous dry-season activity which is the responsibility of mainly young men, but it is also common to see older youths of both sexes involved (Coppock 1994). The watering sites are usually visited by large numbers of camels and other animals at a time from the surrounding as well as from distant areas. Mostly the pond and river water sources are shared by wild animals. Such a state of affairs creates a perfect condition for disease spillover, transmission and spread among animals and to humans.

## Types of camel populations in Ethiopia

Camel breed characterization studies are scanty as only few researchers attempted to phenotypically describe Ethiopian camel populations and classify them into distinct ecotypes. Molecular characterization is completely lacking. As with other livestock species, names ascribed to camel populations in Ethiopia usually reflect the area where they are kept or the tribe/clan who keeps them rather than their distinct attributes in terms of phenotype, genetic makeup and/or performance potential. A breed is defined as a population or group of animals having common origin and similar identifying characteristics that distinguish them from another population of the same species. It refers to a race of animals within a species that tend to transmit those identifying characteristics with reasonable consistency.

Tezera and Belay (2002) identified two types of camels, the Agoweyn and Ayuune, reared by the Somali pastoralists based on physical attributes (body size, coat colour), production performance (milk, lactation length, loading and speed, breeding), physiology and behaviour (ability to withstand water deprivation, disease tolerance, feeding behaviour) and geographic distribution. Tefera and Abebe (2012) classify camels generally into four groups identified by their coat colour, conformation and production performance as milk, meat, dual purpose and baggage camels. More recently, Tadesse et al. (2014a, 2015a, b) classified camels in Afar and Somali into seven subpopulations (Table 1). The authors indicated that Jijiga and Hoor camel populations are milk types whereas Liben and Gelleb camel populations are meat type as evidenced by some quantitative and qualitative descriptors.

**Table 1:** Camel ecotypes in Ethiopia

Camel type	Geographic distribution	Colour and hair type	Conformation	Hump position	Remarks
Jijiga camel	7° 10′ N to 9° 30′ N and 42° 00′ E to 43° 15′ E. Found in Jijiga and Fik zones of Somali Region	Predominantly brown colour, medium hair length	Predominantly large udder and teats, large milk vein and abdomen; milk type animal conformation of triangular shape	Thoracic	During dry season, they migrate 20 to 60 km to Kora, Daketa and Gobayle within their breeding tract
Hoor camel	5° 15′ N to 6° 44′ N and 43° E to 44° 16′ E. Found in Gode, Afder and Kebridahar zones of Somali Region	Varies from brown to red and yellowish white, short hair length	Small ear, large udder and teat size, long tail and large abdominal girth	Thoracic	Preferred because of their high milk production potential than other population in the breeding tract but considered less resistant to diseases, water scarcity and drought. During dry season, the pastoralists together with their livestock migrate 50 to 100 km to Danan, Afder and Suben
Gelleb camel	5° 15′ N to 6° 44′ N and 43° E to 44° 16′ E. Found in Gode, Afder and Kebridahar zones of Somali Region	Dominant brown and red coat colour, pigmented skin, muzzle and hoofs	Longer in height, long tail, exceptionally wider hip and chest and long chest depth	Thoracic and cervico-thoracic	A crossbred between Hoor and Gelleb camel population is termed *Aiden.* It is said to be more tolerant to high temperature and scarcity of feed and water and resistant to disease than the two parents
Amibara camel	8° 58′ to 10° 00′ N and 40° 5′ to 40° 27′ E; from Awash to Gewane in the north and Bure-Mudaitu and Afambo in the east and west, respectively	Brown to grey coat colour, short hair length	Medium-sized udder and teats, smaller body size and weight and small abdominal and heart girth	Thoracic and cervico-thoracic	During trypanosomiasis infestation period and flooding of Awash River, the pastoralists together with their livestock migrate 30 to 50 km to the highland around Argoba area. During dry season and drought period, they migrate 50 to 200 km up to Shewa-Robit, Mollale, and keep their livestock adjacent to Awash River
Mille camel	11° 9′ to 13° 43′ N and 40° 25′ to 41° 22′ E. Distributed in areas from Mille to Chifra to the West and Dubti to the North	Red to brown coat colour with short hair length	Medium to long tail, small body size, large ear, long neck and long legs are the main features of this population; medium udder and teat sizes	Thoracic	During dry and drought periods, migration distances reach 100 to 250 km to zone 4 (Yalo and Teru districts) and zone 5 (Dalifage and Dawe districts) of Afar region and up to Bati in Amhara region
Liben camel	3° 30′ to 5° 30′ N and 39° 00′ to 41° 00′ E (in Liben and Borana zones)	Brown, red, black and white	Large ear size, large hoof circumferences with long legs, heavy body weight, large heart and abdominal girth, with wide hip and chest		Considered to be meat type animal. During dry/drought periods and occurrence of conflict, the population migrates 100 to 200 km to Konso and Gofa districts in SNNPR
Shinille camel	9° 30′ to 10° 30′ N and 41° 15′ to 42° 30′ E (Shinille Zone and eastern Oromia region)	Grey and brown	Short neck and large ears, small body size and light weight, muscled and prominent shoulder and rump, large udder and medium teat size	Thoracic	Appropriate to pull and carry heavy equipment, known for its aggressive character. During dry and drought periods, the camel population migrates 50 to 100 km out of their breeding tract to Chelenko, Daketa and Fafen in Jijiga area

Adapted from Tadesse et al. (2014a, 2015a, b)

## Husbandry practices

### Camel holding

Average camel herd sizes per household among different camel-keeping societies in Ethiopia are summarized in Table 2. Ownership usually varies from several hundreds, 50 to 100 and less (5 to 10) camels. Females are found to be numerically dominant, mostly accounting for above 75% of the herd. Male camels are usually sold early as pack animals or for slaughter.

**Table 2:** Average camel holding per household in major camel-rearing areas

Region	Location	Camel holding per household		Source	Remarks
		Average	Range		
Somali	Jijiga	37.6	-	Tezera and Belay 2002	1989 estimate
	Jijiga	35.2	7 to 93	Tezera and Belay 2002	1996 estimate
	Jijiga	20.4	4 to 40	Tadesse et al. 2014a	
	Jijiga and Shinille	25.7	1 to 150	Eyasu 2009	
	Shinille	26.1	-	Tezera and Belay 2002	1989 estimate
	Shinille	22.7	4 to 73	Tezera and Belay 2002	1996 estimate
	Shinille	20.2	2 to 35	Tadesse et al. 2014a	
	Gode	27.5	6 to 52	Tadesse et al. 2014a	
	Moyale	24.1	8 to 50	Tadesse et al. 2014a	
	Babile	34.5	16 to 66	Sisay et al. 2015	
	Gursum	28.5	16 to 51	Sisay et al. 2015	
Afar	Amibara	19.2	4 to 50	Tadesse et al. 2014a	
	Mille	28.1	2 to 35	Tadesse et al. 2014a	
Oromia	Borana	19.6	-	Megersa et al. 2008	
	Borana	13.33		Dejene 2015	

There seems to be inconsistency regarding reports on trends of camel herd size per household. Increasing trends in ownership of camels by Borana herders (Desta and Coppock 2004) and the Issa-Somali in Shinile zone (Kassahun et al. 2008) were previously reported, and they attributed the increases to drought resistance qualities of camels, changing vegetation and other factors. However, comparing the number of camels per household estimated in 1989 (Tezera and Belay 2002) and some 15 years later (Tadesse et al. 2014a) for Jijiga and Shinille zones of Somali region, one can see a decline, on average, by about 13.2 and 5.9 heads, respectively (Table 2). Similarly, there is a difference between herd sizes reported for Borana area in 2008 and 2015 (a decline by 6.27). The inconsistency may be attributed to differences in sampling the study targets or actual decline in holding per household. It can also be due to the recent boom in camel offtake from pastoralist areas which might deplete animals, though pastoralists reportedly attempt to shift their herd compositions to produce more camels for the market (Aklilu and Catley 2010).

### Feeding management

Traditional pastoral livestock production in Ethiopia is characterized by individual stock ownership, communal use of pastures and seasonal migrations of herds and households. The frequency of migrations might range from once to as much as six times per year, and migration distances might be very short or extend over 200 km (Schwartz and Walsh 1992). In the semi-arid or arid lowlands with a very sparse vegetation growth composed of bushes, trees, shrubs and grasses, camels are subjected daily to travel 14 to 20 km away from their village in search of feed (Wolde 1991; Ahmed Shek et al. 2005a, b). Camels feed mainly by browsing on trees or bushes, but they also graze grasses when these are not available. Usually, there is no supplementary feed provided except salt every two to three months (Wolde 1991). Dromedaries take as much as 90% of their diet under semi-natural conditions from those browse plants. Explained proportionally, this is more than that taken by goats from browse species. An important feature of camels’ browsing habits is that they are not in direct competition with other domestic stock either in terms of the type of feed eaten or in the height at which they eat above the ground (Wilson 1989).

Under open-range conditions, camels tend to move rapidly from one feeding location to the next and they are thus able to exploit a wide variety of plants and of plant parts. Ingestion rates can be rapid where preferred or selected browse is plentiful but is much slower on thorny species that have little leaf. Feeding times required may be as much as 15 or more hours per day, as studies have shown that total dry matter intake needs to be about 4% of body weight (Wilson 1989). A mature dromedary weighing 650 kg would thus require 25 kg of dry matter, which might represent 80 to 100 kg of total feed intake of plants with high moisture content. Camels can achieve these amounts of intake only if they are not required to do much walking to and from the grazing area. Working camels obviously do not get the amount of time required for feeding to satisfy the total feed intake. Camels can overcome this problem, provided work is not continuous, by eating in excess of their immediate needs and storing the extra as fat in the hump (Wilson 1989).

Dromedary camels are extremely efficient at ‘storing’ water because of their physiological, anatomical and behavioural adaptations and not because their humps contain large quantities of water as was assumed in the past. Their efficiency in conserving water is, however, in inverse proportion to the use they are allowed to make of these adaptations; imposition of work or other forms of stress greatly reduce their ability (Wilson 1989). According to the author, the major mechanism of the camel in conserving water is body temperature which may rise by as much as 7 °C during the day. This reduces the need to shed the heat load by sweating or panting. Excess heat is dissipated in the cooler night temperatures without loss of water. By this and other methods, camels can go not only for the commonly quoted four to seven days without water, but on occasions for several months, especially when plants with high moisture content are eaten (Wilson 1989). Ahmed Shek et al. (2005a, b) observed adult camels could survive without water for 44.6 days with mean feed deprivation tolerance of 31 to 39 days and that camels were the last species to be taken to market in Somali region during a drought.

Camels may be watered every 10 to 15 days if the water source is nearby (Wolde 1991; Bekele and Kibebew 2002) but only once in 30 days if the source is far away. However, Ahmed Shek et al. (2005a, b) recorded shorter mean watering intervals of 6.7 to 7.2 days during the dry season in Afder zone, Somali region. During the rainy season, camels may not drink water for one to two months and depend only on the moisture content of the plants browsed. The amount of water camels can consume at a time has been estimated inconsistently: as much as 200 l (Wolde 1991); 90 l in a very short time following severe dehydration amounting to 30% of initial body weight (Wilson 1989); 126 to 140 l at first pause; and 49 to 55 l at the second pause (Ahmed Shek et al. 2005a, b).

Mobility is an inherent strategy of the pastoralists to efficiently utilize the spatially and temporally distributed grazing and water resources. Camels and cattle usually trek over long distances in search of feed and water. The herd may be subdivided in to what is known as ‘wet herd’ and ‘dry herd’. The wet herd is composed of milking cows (of both cattle and camels) and their calves that are kept around homesteads. The dry herd travels long distances. In Borana, for instance, the grazing lands are thus sub-divided into dry- and wet-season grazing, accordingly (Coppock 1994). However, among the Borana and Guji who were primarily cattle herders and who used to highly value cattle more than camels, utilization of rangelands by camels is restricted, i.e. cattle herders restrict camels from passing or browsing grazing lands before cattle. In addition, the hierarchy for camels to get access to water is after cattle (Megersa et al. 2008).

In the case of camels, about 90% of the Karayu Oromo migrated to other distant districts inhabited by other Oromo clans and trek them as far as 250 km (as far as Siraro and Shala in the south or Ada’a/Dukem areas to the northwest approaching the capital Addis Ababa very closely) from their centre (Gebru et al. 2008). Many of the Afar pastoralists in Awash Fantale and Amibara districts (about 98%) migrated with their animals to Samayu, Madal, Fantale, Bulga riverside, near the hot spring. However, the overwhelming majority of both ethnic groups (90% of the Karayu Oromo and 60% Afar) were worried that mobility as a strategy has already become a source of concerns around security of their animals and their lives, death of animals *en route*, incidence of diseases and predators (Gebru et al. 2008).

In the Somali region, 95% of the pastoralists practise traditional nomadic and transhumance management systems whereas only 5% of them are sedentary pastoralists. The mobility pattern involves taking camels to mountain areas during wet season to avoid tick infestation and flies (hence to prevent tick-borne diseases and foot wounds) and to valleys during dry season in search of cactus (*Opuntia ficus indica*) and water for their camels (Keskes et al. 2013). It is a common practice for Somali camel keepers to cross national and international boundaries in search of feed and water particularly during drought years. In Jijiga, many households (58%) practise agro-pastoralism while the other 42% practise pastoralism (Keskes et al. 2013).

### Housing

Camels are usually herded during the daytime on communal grazing lands and kept during night in traditional *kraals* made of thorny bushes and tree branches around homesteads or settlements as protection from predators and thieves/raiders. It is not a common practice to keep camels with other species in a single *kraal*. In some areas, camels may also be left to roam around during night time.

### Camel breeding

Definition of breeding objectives (BO) for specific production systems is extremely important. BOs are clear and concise statements of high-level goals or targets that are production system specific. They may include all relevant attributes of an animal with defined and tangible economic values (e.g. production, reproduction, fitness and health characteristics) and intangible values such as aesthetic virtues of an animal. The importance of each attribute depends on production circumstances (e.g. milk vs. meat or fitness vs. other reproduction traits). Vigilant analysis of information on all aspects of the production system provides a set of BOs (FAO 2010).

Studies on definition of BO traits of camel keepers in Ethiopia are too scanty. Limited available literature indicates that pastoralists highly value and consider milk production potential of camels as evidenced by their trait preferences (Tadesse et al. 2014b), proportion of female camels they keep in the herd (Megersa et al. 2008; Ahmed Shek et al. 2005a, b) and their bull selection practices (Wolde 1991; Tezera and Belay 2002). It is a common practice for pastoralists to keep higher number of female camels than males at all age categories, i.e. calves, growing young ones and adults (Tezera and Belay 2002; Bekele and Kibebew 2002), indicating the importance of reproduction and milk production in arid areas.

Tadesse et al. (2014b) reported that pastoralists in Amibara, Mille, Shinille, Gode, Liban and Jijiga ranked milk yield as their first trait of choice; for those in Moyale district, adaptation trait was the primary preference. The authors further indicated growth trait was ranked second by Mille, Gode, Liben and Jijiga pastoralists whereas adaptation trait was ranked second by Amibara and Shinille pastoralists. Trait preference indices also revealed milk production ranked first with adaptability, breeding efficiency, growth, ability to give birth to more female and draught capacity traits with changes in rank across the different sites (Tadesse et al. 2014b).

Usually, a breeding bull is selected on criteria such as colour, beauty, size, physical condition and the milk production potential of its ancestors (Wolde 1991; Tezera and Belay 2002). In addition, preference is given to a bull that hails from more female-bearing ancestors. This type of bull is believed to have a shiny hair coat and on the whole look like a ‘beautiful dam’. If the selected breeding bull produces a higher proportion of male offspring for three consecutive years, it is culled and replaced by a new bull (Tezera and Belay 2002). Once a bull is selected as a stud, it is not used for any purpose other than breeding until the end of its reproductive life (Wolde 1991; Keskes et al. 2013a, b). Male camels which are not fit as stud are either culled or separated from the herd and tamed for draught (Wolde 1991). On the other hand, female camels are not usually culled except due to reasons such as diseases, old age and poor (re) production performances (Keskes et al. 2013b). Farah et al. (2004) report similar breeding management practised by pastoralists in northern Kenya who also focus on the selection of breeding bulls employing specific criteria which may include a bull’s dam (milk production, fitness), bull’s sire (fitness) and a bull’s own performance (body confirmation, fitness, docility and disease and drought tolerance).

There is considerable divergence regarding the practice of allocating breeding females to a breeding male (i.e. the male to female ratio) among the Ethiopian camel breeders. For instance, those in Ogaden select only one stud bull for a herd of 40 to 50 females (Wolde 1991). Similarly, in Afar, only one bull is assigned for a herd of 30 to 50 females (Keskes et al. 2013a). On the other hand, 2 to 4 bulls may be kept in the herd with a male to female ratio of 1:13 in Shinille, Jijiga and Moyale districts (Tadesse et al. 2014b). There is also a considerable difference as to the ideal male to female ratio during the breeding season. According to a review by Mukasa-Mugerwa (1981), estimates vary from as low as 1 male per 5 to 7 females, through medium levels of 1:10 to 30 to as high as 1:50 to 80. Major determining factors include the management practices of pastoralists, the condition and stamina of the male, his libido and the fertility level of the females. A bull can breed three females per day at the peak of the breeding season, although higher levels are possible (Mukasa-Mugerwa 1981).

Among some camel producers in Eastern Africa, it is a common practice to forcefully mate female camels whenever a rutting bull is available and may result in over 50% successful conception (Schwartz and Walsh 1992). Usually, ovulation in camels can be induced by mating, artificial insemination and spontaneously during the height of the breeding season as their oestrous cycle consists merely of follicular waves, that is a continuous development and regression of follicles during the breeding season. Ovulation takes place 36 to 48 h after the stimulus. Multiple ovulations are possible, but the incidence of twin birth is very low. There is evidence for high early embryonic loss rate. The gestation period is about 13 months (370 to 390 days) long (Schwartz and Walsh 1992).

It is common for camel-herding men to aid the entrance of the male penis into the female genitalia, although experienced breeding bulls can often mate the females without help (Mukasa-Mugerwa 1981; Tezera and Belay 2002). However, inexperienced bulls, which are either less than six or seven years old, or which were previously used for transport and then for breeding, mostly need assistance, usually provided by male herders during mating (Tezera and Belay 2002).

Even though camels in the tropics are not generally seasonal breeders, camels in Ethiopia show a seasonal reproductive function, breeding (mating) and calving patterns which correspond to the annual/biannual rainfall pattern and feed availability. In Borana, the major breeding and calving season extends from April to June while a minor breeding season occurs during October and November (Megersa et al. 2008). Somali camels around Jijiga and Shinille are bred mostly during the wet season between April/May and September (Tezera and Belay 2002). In Erer valley, East Hararghe, Mekuriaw and Tafesse (2000) monitored pastoral herds and found that 85% of the matings and 86% of the calvings occurred during the wet season, demonstrating the seasonality of reproduction among camels in the area. These observations are in congruence with the report of Schwartz and Dioli (1992) that the breeding season of camels (both males and females) is very short and coincides with the rainy season, implying their reproduction performance is influenced by nutritional factors.

### Milking

Milk extraction for human consumption begins three days after calving. Following stimulation of milk let-down by a suckling calf for few seconds, milk is extracted by hand into a milking vessel, commonly a wooden container. Only males are allowed to milk camels among the Afar, Boran and Somali pastoralists (Tafesse et al. 2002; Eyasu 2009; Keskes et al. 2013; Sisay et al. 2015; Tadesse et al. 2015a, b). Among the Afar and Somali pastoralists in particular, women are not allowed to milk camels because camels are highly valued and considered as sacred animals among both societies (Tadesse et al. 2015a, b). In addition, as camels are milked in a standing position, the task requires enormous energy which makes it difficult for females to perform milking camels. Besides there is a belief held by the communities that lactating camels do not allow women to milk them or do not let down sufficient milk for women milkers (Tadesse et al. 2015a, b).

In the typical milking routine, as described by Eyasu (2009), the owners prepare a milking vessel and call a lactating camel by name from the enclosure to a separate open milking area where the calf is kept. Then, the calf is allowed to suckle its dam for a few seconds to around a minute to stimulate milk ejection. After this, one man holds or chases the calf away while another man milks the camel at a standing position with one knee raised to support the milking vessel on his lap. It is also common for two men standing on opposite sides of the camel performing milking simultaneously, each working on the right and left quarters of the udder (Tafesse et al. 2002). Milking frequency ranges from twice to five times a day (Eyasu 2009; Sisay et al. 2015). Washing of the udder and teats of the camels before milking is not practised by many pastoralists, and they do not wash their hands and the milking vessels prior to milking. The milking area is generally full of dust and dung and without shade, causing a negative impact on the quality and safety of the milk produced (Eyasu 2009).

## Production performance

Reports of camel productive performance evaluation studies in Ethiopia under controlled experimental conditions are not available. Evaluation studies are mostly based on the monitoring of pastoral herds managed traditionally or on producer memories extracted through interviews. Table 3 presents the estimates of productive performance of Ethiopian camel populations summarized from various studies.

**Table 3:** Estimates of productive performance of Ethiopian camel ecotypes

Parameter	Estimates	Location	Source
Milk yield, kg/day	8 to 10	Ogaden, Somali	Wolde 1991
	2.92 (2.7 to 4.92)	Afar and Somali	Tadesse et al. 2015a, b
	4.14 (1.26 to 6.77)	Erer, Somali	Bekele et al. 2002
	5.2 (1 to 10)	Jijiga and Shinille, Somali	Eyasu 2009
	6	Borana	Megersa et al. 2008
	4 (3 to 5)	Jijiga, Somali	Sisay et al. 2015
	6.57	Borana, Oromia	Dejene 2015
	3.75	Erer valley, East Hararghe	Mekuriaw 2007
Milk yield, kg/lactation	2009	Jijiga, Somali	Tezera and Belay 2002
	1244	Shinille, Somali	Tezera and Belay 2002
	1585	Afar and Somali	Tadesse et al. 2015a, b
	1422	Erer, Somali	Bekele et al.^ 2002^
	2040	Babille and Gursum, Somali	Sisay et al. 2015
Lactation length, months	14	Ogaden, Somali	Wolde 1991
	13.76 (10.75 to 19.4)	Afar and Somali	Tadesse et al. 2015a, b
	11.51 (6 to 24)	Somali	Keskes et al. 2013b
	12	Afar	Keskes et al. 2013a
	12 (7.5 to 18.9)	Erer, Somali	Bekele et al. 2002
	12.7 (6 to 24)	Jijiga and Shinille, Somai	Eyasu 2009
	13.85	Babille, Somali	Sisay et al. 2015
	12.53	Gursum, Somali	Sisay et al. 2015
	13.38	Borana, Oromia	Dejene 2015
Growth rate, g/day	222	North Kenya	Yusuf and Bekele 2004
	50.68	Erer valley, East Hararghe	Mekuriaw and Tafesse 2000
	655	Experimental condition	Yusuf and Bekele 2004
Offtake, %	3.7	Borana, Oromia	Megersa et al. 2008
	4.86	Afar Region	Keskes et al. 2013
	4.74	Erer valley, East Hararghe	Mekuriaw and Tafesse 2000
Dressing percent	52.8	Isa camel, Somali	Wolde et al. 2002
	54.03 ± 5.13	Eastern Ethiopia, male camels	Kurtu 2004
	50.65 ± 3.70	Eastern Ethiopia, female camels	Kurtu 2004
Carcass yield, kg	233.4	Isa camel, Somali	Wolde et al. 2002
	230.02 to 240.28	Babile, male camels	Mehari et al. 2007
	187.74 to 195.14	Babile, female camels	Mehari et al. 2007
	214.77 to 225.03	Kebribeyah, male camels	Mehari et al. 2007
	199.76 to 207.16	Kebribeyah, female camels	Mehari et al. 2007
Herd growth, %	0.3 to 18.6	World estimate	Yusuf and Bekele 2004
	10.66	Ethiopia estimate	Yusuf and Bekele 2004

### Growth and weaning

Camels are known to be slow-growing and maturing animals. Anecdotal evidences based on on-farm monitoring of pastoral herds in Erer valley (eastern Ethiopia) revealed that growth rate of monitored growing camels was as low as 50 g/day (Mekuriaw and Tafesse 2000). On the other hand, Yusuf and Bekele (2004) quote reports on herds of camels maintained in northern Kenya and those under experimental conditions in unspecified locations as having growth rates of 222 and 655 g/day, respectively (Table 3).

Camel calves are usually weaned at an age of six to 12 months in Eastern Africa, depending on abundance of milk (Schwartz and Dioli 1992). In Ethiopia, mean weaning age, in months, is six with a range of three to nine (Tefera and Abebe 2012). If milk either is in abundant supply or demand is low, the herders may not interfere and let calves suckle until their mothers dry up. Normally, there is stiff competition for the milk between calf and herder, meaning that the calf is forced to be weaned as soon as it is able to consume sufficient forage. Weaning techniques include transfer of the calf to a different herd; tying off the teat; blocking with udder basket; pushing thorns through the upper lips of the calf from inside and fixed in place with acacia resin; cutting a thin strip from each nostril with skin flaps left hanging; or cutting a thin strip of skin from the nose and tying a bark around it so that it stands upright (Schwartz and Dioli 1992). The latter two techniques make suckling uncomfortable to the calf.

### Milk

Camels produce more milk and for longer period of time than any other milk animal held under the same harsh conditions (Farah et al. 2007). Milk is the most important camel product in arid and semi-arid environments of Eastern Africa, and in this region, camel milk is a valuable food source for humans. Total dry matter content of camel milk ranges from 12 to 15%, protein from 2.7 to 4.5%, fat from 2.9 to 5.2% and lactose up to 5.5% (Schwartz and Walsh 1992). The high content of vitamin C, which may reach 2.9 mg/100 g, is of special importance particularly in areas where food of plant origin is rare. Estimates of milk yields, be the daily yields or lactation yields, differ widely. Reported daily yields range from 3.5 to over 20 l; corresponding annual lactation yields from 800 to over 4000 l. Lactation lengths likewise show a large variation of eight months to almost two years (Schwartz and Walsh 1992). Average daily milk yield estimates for camel populations in different regions of Ethiopia range from 1 to 10 kg while lactation yield estimates are between 1,244 kg for Shinille population and 2,040 kg for Jijiga population (Table 3).

### Lactation length

Lactation lengths can be easily recorded or estimated under any production conditions. Camels are known for their longer lactation periods even in the worst years. Estimates abound for all Ethiopian camel populations. Estimates of mean lactation periods are about one year almost for the entire populations. Values ranging from as low as six months to as high as two years have also been reported (Table 3).

### Meat

Not much solid information is available on camel meat production, whereas estimates abound. Adult camel live weights range, depending on age, sex, breed, nutritional status and stomach fill, from approximately 320 to 750 kg; these weights are reached between five and seven years of age in pastoral production systems. Dressing percentage, as in other herbivores, ranges from 45 to 55%. Meat quality is largely age dependent, and as in other meat animals, good meat is from young slaughter stock. The majority of camels slaughtered are culls, and only a limited number of castrated males are especially raised for slaughter. Camel meat markets and camel meat consumption are, with the exception of Sudan, not very well developed in Eastern Africa, but lucrative export opportunities to Egypt, Libya, Saudi Arabia and the Gulf States do exist. Due to the intrinsically low reproductive rate, camels are not efficient meat producers. Offtake rates of 3 to 5% might already constitute a stress on the population (Schwartz and Walsh 1992).

Although many pastoralists consume camel meat when available, camels are never slaughtered for home consumption of meat except occasionally during festive times, to fulfil cultural obligations such as funerals, wedding ceremonies and religious festivals or when camels are accidentally injured (Wolde 1991; Eyasu 2009). However, camel meat is sold to consumers from butcher shops in towns such as Dire Dawa, Harar and Jijiga (Wolde 1991; Kurtu 2004).

A survey on camel meat productivity and consumption conducted in Jijiga and Harar towns by Kurtu (2004) indicated that the dressing percentage of eastern Ethiopia camels was found to be 54.03 ± 5.13 for male camels and 50.65 ± 3.70 for female camels. Mehari et al. (2007) reported carcass yields of 230 to 240 kg for males and 188 to 195 kg for females in Babile and 215 to 225 kg for males and 200 to 207 kg for females in Kebribeyah.

According to Mehari et al. (2007), 53 and 23.5% of respondents from Babile believe that preserved camel meat can stay unspoiled and safe for consumption after five and 10 years, respectively. Similarly, 50% of respondents in Kebribeyah claimed it can stay unspoiled and safe for consumption after many years.

### Work

There is enormous economic significance of the various forms of services rendered by camels (Wolde 1991). Within Eastern Africa, camels are most frequently used as pack animals and also for riding. Use of camels as draught animals is traditionally practised in some parts of Ethiopia. Elsewhere, occasional use is made of camels for driving oil mills, operating water wheels or drawing irrigation water from deep wells (Schwartz and Walsh 1992). They are widely used for transporting water, firewood, commercial goods, and huts and household goods during seasonal migrations. The technology used is simple to primitive. Pack saddles in the region are usually only a collection of sticks, ropes and pieces of rawhide which need to be refashioned at every loading (Schwartz and Walsh 1992).

Only male camels are used as pack animals. They played a crucial role in contraband trades transporting materials from countries such as Djibouti, Somaliland and Somalia. An adult male can carry ~ 300 kg and cover up to 40 km/day (Wolde 1991). Although it is currently restricted to lower altitude areas due to motorization, the caravan salt trade that would never be possible without the ardent services of camels is still in operation and will be remembered for its significant role in the economic and political history of Ethiopia (Aklilu and Catley 2011).

## Reproductive performance

Population growth estimates derived from various simulation models lie between 1.5 and 8% annual increase provided there is no drought, disease outbreak or any other calamity. There is only a marginal scope to improve productivity in the camel via interventions that target any reproduction parameters. The inherent reproductive rate in camels is very low in comparison to all other domestic herbivores, and significant improvements are conceivable only at high cost or will be counterproductive on other productivity parameters (Schwartz and Walsh 1992).

### Puberty and age at first calving

Table 4 gives estimates of some reproductive parameters for Ethiopian camel populations in different areas. Generally, it appears that females reach puberty well ahead of their male counterparts. Age at which female and male camels achieve puberty ranges between 3.9 and 4.7 years and 5.5 and 6.5 years, respectively. The lowest and longest reported age at first calving was 4.95 and six years, respectively (Table 4). On average, female camels deliver their first offspring around five years under Ethiopian conditions. Traditionally managed bulls reach age at first service at 5.5 years and achieve full sexual maturity at seven years of age during which time they achieve the capacity to accomplish 11 services per day and also successfully breed 60 to 70 cows in a breeding season (Ahmed Shek et al. 2005a, b).

**Table 4:** Estimates of reproductive performance of camels in different areas of Ethiopia

Parameter	Estimates	Location	Source
Age at first mating (male), years	6.2	Jijiga, Somali	Tezera and Belay 2002
	6.5	Shinille, Somali	Tezera and Belay 2002
	5.5	Afder, Somali	Ahmed Shek et al. 2005a, b
Age at first mating (female), years	4.7	Jijiga, Somali	Tezera and Belay 2002
	4.4	Shinille, Somali	Tezera and Belay 2002
	3.9	Afder, Somali	Ahmed Shek et al. 2005a, b
	3.97	Somali	Keskes et al. 2013a
Age at first calving, years	5.0 to 5.4	Afder, Somali	Ahmed Shek et al. 2005a, b
	4.95		Tadesse et al. 2015a, b
	6	Somali	Bekele and Kibebew 2002
	5.18	Somali	Keskes et al. 2013a
	5.36	Somali	Keskes et al. 2013a
	5	East Africa	Schwartz and Walsh 1992
	4.87	Somali, North Kenya	Kaufmann 2005
	5.25	Rendille, North Kenya	Kaufmann 2005
	5.7	Gabra, North Kenya	Kaufmann 2005
Lifespan (male), years	22	Jijiga, Somali	Tezera and Belay 2002
	23	Shinille, Somali	Tezera and Belay 2002
Lifespan (female), years	29.8	Jijiga, Somali	Tezera and Belay 2002
	29	Shinille, Somali	Tezera and Belay 2002
Productive life (male), years	9.3	Jijiga, Somali	Tezera and Belay 2002
	9.7	Shinille, Somali	Tezera and Belay 2002
	7 to 10		Tadesse et al. 2014b
Productive life (female), years	23.1	Jijiga, Somali	Tezera and Belay 2002
	22.4	Shinille, Somali	Tezera and Belay 2002
	18.49		Tadesse et al. 2014b
	17.5	Babile/Gursum	Sisay et al. 2015
Number of calves in a lifetime	11.6	Jijiga, Somali	Tezera and Belay 2002
	11.7	Shinille, Somali	Tezera and Belay 2002
	8		Bekele and Kibebew 2002
	9.17	Somali	Keskes et al. 2013a
Calving rate, %	54.6	Jijiga, Somali	Tezera and Belay 2002
	42.5	Shinille, Somali	Tezera and Belay 2002
	42.72	Erer valley, East Hararghe	Mekuriaw and Tafesse 2000
	40.5	Somali	Bekele and Kibebew 2002
	39.6	Borana, Oromia	Megersa et al. 2008
Calving interval, months	23 to 24	Somali	Ahmed Shek et al. 2005a, b
	19.1	Jijiga/Shinille, Somali	Bekele and Kibebew 2002
	23.28	Somali	Keskes et al. 2013b
	24.25	Babile/Gursum	Sisay et al. 2015
	31.2	Afar	Keskes et al. 2013a
	25.5	Borana, Oromia	Megersa et al. 2008
	17.73	Borana, Oromia	Dejene 2015
	28.4	Somali, North Kenya	Kaufmann 2005
	27.3	Rendille, North Kenya	Kaufmann 2005
	28.0	Gabra, North Kenya	Kaufmann 2005
Number of services per conception	1.73		Tadesse et al. 2015a, b
	1.84	Afar	Keskes et al. 2013b
	1.63	Somali	Keskes et al. 2013a
	1.36	Erer valley, East Hararghe	Mekuriaw and Tafesse 2000
Mortality	7.4	Borana, Oromia	Megersa et al. 2008
	15.8 (total)	Afar	Keskes et al. 2013
	12.3 (calf)	Afar	Keskes et al. 2013
	20.42	Erer valley, East Hararghe	Mekuriaw and Tafesse 2000

### Oestrous and calving interval

After reaching sexual maturity, the female dromedary in Eastern Africa exhibits regular oestrous cycles, which nevertheless seem to be limited to particular periods of the year (Mukasa-Mugerwa 1981), mainly influenced by the nutritional status of the animal rather than by photoperiodic phenomenon. Once in breeding age, female camels cycle every 20 to 25 days (average 23.4 days). During the oestrous period, they show both anatomical and nervous signs of heat such as restlessness, seeking company of the male, continuous bleating and developing a swollen vulva often associated with a discharge. The female in heat may emit a penetrating foul smell from the vulva that could be smelt over long distances but which has an excitative effect on breeding bulls (Mukasa-Mugerwa 1981).

Calving interval is normally two years on average (Table 4) with values ranging from close to 18 months in Borana (Dejene 2015) to 31.2 months in Afar (Keskes et al. 2013a).

### Longevity and lifetime productivity

Female camels can remain fertile to an age of 25 years during which time they may produce 8 to 10 calves in a lifetime; in pastoral production systems, only a small fraction of the breeding females will reach this and the average lifetime performance will be around 6 to 7 calves (Schwartz and Walsh 1992). However, Tezera and Belay (2002) recorded 11.6 calves produced by a single female based on herders’ recollection (Table 4).

## Major camel diseases

Pathogens and diseases related to camelids are less well known than those of other domesticated species, but have attracted growing attention recently. For instance, several unusual disease incidents caused by *Trypanosoma evansi* and morbillivirus infection, causing high morbidity and/or mortality rates in camels, were reported in the literature. There is an increasing need to determine whether camels are clinically susceptible and act as potential reservoirs and maintenance or bridge hosts to viral pathogens affecting other livestock and/or humans. Overall, dromedaries seem to be more resistant hosts for bovine, ovine or caprine viral diseases such as foot-and-mouth disease or rinderpest (Miguel et al. 2016).

Trypanosomiasis, camelpox, contagious ecthyma, dermatomycosis, pneumonia, mange mite infestations and internal parasites are among the major health problems previously reported in camels in Ethiopia (Richard 1979; Demeke 1998; Tefera and Abebe 2012). Camels may also be susceptible to bovine viral diarrhoea, infectious bovine rhinotracheitis, parainfluenza-3, respiratory syncytial virus and Rift Valley fever (Odeh et al. 1999; Brown 2004). Of the many diseases of camels that are rampant in the country, the 2006/2007 outbreak with hitherto undetermined cause(s) is the single most important disease with huge mortality (Tefera and Abebe 2012). The outbreak, however, seems to have been caused by a PPR-related virus (Roger et al. 2000; Haroun et al. 2002; Abraham et al. 2005; Khalafalla et al. 2010).

In Erer, Somali Region, trypanosomiasis was one of the most important diseases identified with a maximum prevalence of 20.6% and minimum of 5.4% followed by Sarcoptes mange mite lesions, strongyle parasites and ticks that were prevalent throughout the year but with higher prevalence during rainy months than dry months (Mekuriaw and Tafesse 2000). Mohammed et al. (2015) also found a prevalence rate of 8.1% for *Trypanosoma evansi* in camels in Babile, East Hararghe. The prevalence rate of sarcoptic mange mites varied from 21.7 to 4.7% during rainy and dry months, respectively. The highest prevalence rate of strongyle eggs was 85.7% during rainy months and the lowest was 61.5% during dry months (Mekuriaw and Tafesse 2000). The authors observed during their monitoring period two outbreaks of camelpox that affected only young animals from six months to two years of age. They also noted the occurrence of a new camel respiratory disease that affected almost 85.8% of the monitored animals (Mekuriaw and Tafesse 2000). Keskes et al. (2013b) noted that the most prevalent diseases in Somali area were camelpox (51%), anthrax (29%), trypanosomiasis (10%) and respiratory diseases (4%).

In Afar, respiratory tract problems and external parasite infestations were the major diseases followed by trypanosomiasis, brucellosis and internal parasite infestation (Keskes et al. 2013a). Camel trypanosomiasis was reported as an important single cause of economic losses in Ethiopia, causing morbidity of up to 30% and mortality of around 3% in camels (Mohammed et al. 2015).

## Dromedary camels and MERS-CoV

Dromedary camels are strongly suspected of acting as a zoonotic source for human cases of MERS-CoV, by either direct contact through droplet infection via mucous membranes or indirect contact through milk, meat or urine. In review of available literature on MERS-CoV, Miguel et al. (2016) present five major accounts that suggest dromedary camels can play an important role in the epidemiology of the disease, possibly as a reservoir host: (1) coronaviruses are widespread in the animal kingdom (in bats and livestock), but MERS-CoV does not infect many of the hosts (e.g. sheep, goats, cattle, chickens, water buffaloes, birds, horses and other camelids such as llamas, alpacas and Bactrian camels) whereas high levels of seroprevalence have been observed in dromedary camelids, ranging from 0% in Asia to as much as 100% in Africa and the Arabian Peninsula (with mean of 79%); (2) the MERS-CoV isolated from dromedaries are genetically and phenotypically very similar or identical to those infecting humans; (3) retrospective serological studies in Africa going back more than 30 years indicate long-term circulation of the virus in dromedary camels; (4) infection in dromedaries causes no or only mild respiratory symptoms, making it difficult to detect; (5) MERS-CoV genome has likely undergone numerous recent recombinations, which suggests frequent co-infection, probably in camels, with distinct lineages of MERS-CoV. Similarly, Reusken et al. (2014) found high percentages of animals sampled from Nigeria and Ethiopia being seropositive for MERS-CoV with an overall seropositivity of 94% in adult dromedaries in Nigeria and 93% and 97% for juvenile and adult animals, respectively, in Ethiopia. More recently, Miguel et al. (2017) found a high seropositivity of 99.4% in Ethiopia and also relatively higher MERS-CoV RNA detection in Ethiopia (15.7%) than in Burkina Faso (12.2%) and Morocco (7.6%). Fekadu et al. (2017) also reported 93% seropositivity and 7% MERS CoV RNA detection in Ethiopian camels.

However, data from experimental camel infections conducted in the Middle East suggest that MERS-CoV causes only mild respiratory infection in camels (Adney et al. 2014) despite high levels of seropositivity. It has also been observed that camel calves found shedding MERS-CoV in natural field settings did not have overt clinical symptoms (Hemida et al. 2015a, b; Wernery 2014; Wernery et al. 2015). Furthermore, virus-shedding adult camels sampled at abattoirs did not have overt MERS-CoV clinical symptoms (Hemida et al. 2015a).

Critical knowledge gaps abound around this new disease. However, some studies have demonstrated that dromedary camels can act as a source of human MERS-CoV infection. Indeed, the current state of knowledge indicates that dromedary camels are the only animal species for which there is convincing evidence that they act as host species for MERS-CoV and hence a potential source of human infections. Nonetheless, the route of infection and types of exposures remain largely unknown, and only a small proportion of the primary cases have reported contact with camels. Other possible sources and vehicles of infection include food-borne transmission such as unpasteurized camel milk and raw meat, and medicinal use of camel urine (Memish et al. 2014a, b; Gossner et al. 2016). Clearly, transmission from camels to humans does take place, and camel exposure is a risk factor for human infection, but such transmission is not efficient and infection is not directly proportional to exposure. Many thousands of people in the Arabian Peninsula appear to have evidence of unrecognized past MERS-CoV infection. On the other hand, many patients with clinically diagnosed MERS did not have an obvious history of direct exposure to camels or their products (Hemida et al. 2015a). Although dromedary camels are strongly suspected of acting as a zoonotic source for human cases of MERS-CoV, most documented infections originate from medical institutions or, to a lesser extent, within households (Miguel et al. 2016). Thus, the exact role of camels as a reservoir for MERS-CoV is still unclear.

## Conclusion

A large number of dromedary camels are widely distributed throughout the arid/semi-arid lowland areas of Ethiopia predominantly inhabited by pastoral and agro-pastoral communities. They are indispensable for the livelihoods and survival of the majority of the pastoralists in these areas. Their role as one of the most important livestock species for nutrition in the arid and semi-arid areas of the country, and Eastern Africa in general, is likely to increase due to the increasing impacts of climate change and recurrent droughts.

The fact that camels are used for trade, both in desert caravans and mainly as live exports for the meat market, means that they connect distant human populations and their livestock and hence may play a role in the large-scale dissemination of viruses if the viruses can survive in the host populations during transport. The water resources on which animals and humans rely for drinking are considered hotspots for pathogen transmission and favour the exchange of parasites (virus, bacteria) between hosts. Usually, large numbers of camels and other animals from many different herds/flocks congregate at watering sites, and this may create a perfect condition for disease transmission and spread among animals. The same water sources are also shared by multitudes of wild animals. Other important risk factors include, but are not limited to, migration or mobility over long distances in search of feed and water; breeding practices particularly assisting inexperienced bulls during copulation with bare hands; unhygienic milking; culture of consuming raw milk; use of different camel products (milk, meat and urine) for medicinal purposes; intimate relationship between the camels and herders; and weaning practices such as transfer of the weaner camels to different herds. Extreme climatic events associated with climate change, which cause long periods of drought followed by short but severe downpours or floods, may also have a severe impact on the health of camel populations and of humans and other livestock.

We conclude that the particularities of camels and the specific culture and associated human behaviour involved in their rearing can create transmission pathways linked to the camel-human relationship, offering specific routes of spillover for viruses. In order to understand the roles of camels in the epidemiology of MERS-CoV, comprehensive production systems, value chains and surveillance studies are warranted.

## Abbreviations

BO: Breeding objectives
FAO: Food and Agriculture Organization of the United Nations
MERS-CoV: Middle East respiratory syndrome coronavirus
PPR: *Peste des petits* ruminants
SNNPR: South Ethiopia Nations, Nationalities, and Peoples Region
WHO: World Health Organization
